# Synthesis and Characterization of Biodegradable Polymer Blends Based on Chitosan

**DOI:** 10.3390/polym17131853

**Published:** 2025-07-02

**Authors:** Lyazzat Bekbayeva, Grigoriy A. Mun, Bayana B. Yermukhambetova, El-Sayed Negim, Galiya Irmukhametova, Khaldun M. Al Azzam, Sergey V. Nechipurenko, Sergey A. Efremov, Mubarak Yermaganbetov, Moshera Samy

**Affiliations:** 1 National Nanotechnology Open Laboratory, Al-Farabi Kazakh National University, Al-Farabi Av., Almaty 050040, Kazakhstan; lyazzat_bk2019@mail.ru; 2 National Engineering Academy of the Republic of Kazakhstan, Bogenbai Batyr Str. 80, Almaty 050010, Kazakhstan; mungrig@yandex.ru (G.A.M.); galiya.yrmuhametova@kaznu.edu.kz (G.I.); sergey.nechipurenko@kaznu.edu.kz (S.V.N.); sergey.efremov@kaznu.edu.kz (S.A.E.); 3 Department of Chemistry & Technology of Organic Substances, Natural Compounds and Polymers Al-Farabi Kazakh National University, 71 Al-Farabi Av., Almaty 050040, Kazakhstan; 4 School of Chemical Engineering, Kazakh British Technical University, 106 Walikhanov Street, Almaty 050010, Kazakhstan; a.negim@kbtu.kz; 5 Department of Chemistry, Faculty of Science, The University of Jordan, Amman 11942, Jordan; k.alazzam@ju.edu.jo; 6 Department of Chemical and Biochemical Engineering, Satbayev University, 22 Satbayev Street, Almaty 050013, Kazakhstan; e.mubarak@mail.ru; 7 Polymers and Pigments Department, National Research Centre, 33 El Buhouth St., Dokki, Giza 12622, Egypt; ms.elaziz@nrc.sci.eg

**Keywords:** chitosan, itaconic acid, carbon black, starch, biodegradable, mechanical properties

## Abstract

Despite its broad application due to its affordability, biodegradability, and natural antimicrobial and antioxidant activities, chitosan (CS) still exhibits limitations in mechanical strength and barrier effectiveness. Owing to its unique chemical characteristics, itaconic acid (IT) presents potential as a compatibilizing agent in polymeric blend formulations. Biodegradable polymers composed of chitosan (CS), itaconic acid (IT), and starch (S) were synthesized using two polymerization methods. The first method involved grafting IT onto CS at varying ratios of IT (4%, 6%, and 8% wt.), using 1% *v*/*v* acetic acid/water as the solvent and potassium persulfate as the initiator. In the second approach, starch (S) was blended with the copolymer P(CS-g-IT) at concentrations of 1%, 3%, and 5%, utilizing water as the solvent and glacial acetic acid as a catalyst. The resulting biodegradable films underwent characterization through FTIR, TGA, SEM, and mechanical property analysis. To further explore the effects of combining IT, starch, and carbon black, the blends, referred to as P[(CS-g-IT)-b-S], were also loaded with carbon black. This allowed for the evaluation of the materials’ physicomechanical properties, such as viscosity, tensile strength, elongation, and contact angle. The findings demonstrated that the presence of IT, starch, and carbon black collectively improved the films’ mechanical performance, physical traits, and biodegradability. Among the samples, the blended copolymer with 1% starch exhibited the highest mechanical properties, followed by the grafted copolymer with 8% IT and the blended copolymer mixed with carbon black at 7%. In contrast, the blended copolymer with 5% starch showed the highest hydrophilicity and the shortest degradation time compared to the grafted copolymer with 8% IT and the blended copolymer mixed with 7% carbon black.

## 1. Introduction

In recent years, biodegradable polymers have attracted considerable attention due to their ability to decompose into non-toxic, eco-friendly byproducts [1]. These materials and their composites possess a range of mechanical, thermal, and electrical properties that make them suitable for various applications [2]. Among these, chitosan (CS) stands out as a naturally derived polymer obtained through the deacetylation of chitin. It is primarily composed of 1→4 linked 2-amino-2-deoxy-β-D-glucopyranose (D-glucosamine) units [3]. Recognized for its excellent biocompatibility, biodegradability, and antibacterial nature, CS has emerged as a leading polysaccharide for biomedical uses [4].

Owing to its antioxidant activity and favorable physicochemical properties, CS is extensively applied in sectors such as food processing, agriculture, medicine, and cosmetics [5,6]. To improve its performance and broaden its practical applications, various physical and chemical modifications of CS have been explored, including crosslinking, blending, chemical derivatization, complex formation, and graft copolymerization [7,8]. Among these, blending CS with other polymers—both synthetic and natural—has proven to be a particularly promising approach due to its simplicity, scalability, and flexibility [9]. To develop fully biodegradable polymer blends based on CS, the use of complementary biodegradable polymers is essential. Nevertheless, the relatively poor mechanical strength of pure CS limits its use in certain biomedical applications [4]. This limitation can be addressed by incorporating reinforcing biopolymers such as starch or polyvinyl alcohol (PVA), both of which offer improved mechanical characteristics and enhance the performance of CS-based materials.

Lending chitosan (CS) with starch, a naturally abundant and cost-effective biopolymer, offers promising potential due to starch’s biodegradability and non-immunogenic nature [10]. Starch, especially in the form of thermoplastic starch (TPS), has been widely explored for producing biodegradable films. However, its high hydrophilicity can compromise mechanical stability under humid conditions [11]. Incorporating CS into starch-based films helps mitigate these limitations. This enhancement arises from the strong intra- and intermolecular hydrogen bonding between the amino and hydroxyl groups on the backbones of CS and starch, leading to improved film formation [12]. The properties of the resulting CS/starch blends—such as mechanical strength, water resistance, and compatibility—are influenced by the proportion of each component [13]. These blends often demonstrate superior hydrophilicity, mechanical performance, and biocompatibility compared to films made from individual polymers [14].

Itaconic acid (IT), a naturally occurring substance derived from the fermentation of algae, has been extensively employed to alter natural goods including starch [15,16] and chitosan, alginate, cellulose, and sisal [17,18,19]. IT is classified as a negatively charged, tiny molecular substance and is a dual unsaturated carboxylic acid. Because of its distinct chemical properties, IT may be used as a compatibilizing agent in polymeric blends [20]. Among the many compounds for which IT is used as a building block are resin, paints, plastics, and synthetic fibers (acrylic plastic, super absorbents, and anti-skating agents) [21,22,23]. In the presence of initiators, it also exhibits strong self-polymerization activity. The enhanced properties of chitosan/IT blends are attributed to interactions such as hydrophobic side-chain aggregation and hydrogen bonding—both intramolecular (involving secondary hydroxyl groups and ether oxygen within chitosan) and intermolecular (between the primary hydroxyl groups of chitosan and the hydroxyl groups of itaconic acid). To preserve a useful level of swelling and enhance the mechanical qualities of pure chitosan, the current study aimed to synthesize blended polymers of chitosan and itaconic acid. The novel method was to dissolve chitosan in itaconic acid, which was different from other literature reports. Itaconic acid’s two COO^−^ groups interacted with chitosan’s NH^3+^ ions in acidic environments to create ionic crosslinks between the macromolecules of chitosan [24]. Furthermore, combining CS with both IT and starch creates a ternary blend expected to exhibit desirable mechanical and processing characteristics [25]. The strong hydrogen bonding among hydroxyl groups in IT and starch contributes to the excellent miscibility and performance of these biodegradable polymer mixtures [26,27].

In this study, novel biodegradable polymer composites based on CS, itaconic (IT), and starch were synthesized, incorporating carbon black as a filler. These materials were evaluated for their structural, mechanical, and biological properties. Fourier-transform infrared spectroscopy (FT-IR), thermogravimetric analysis (TGA), and scanning electron microscopy (SEM) were employed to comprehensively analyze the structural and compositional characteristics of the films. The findings aim to support the development of eco-friendly materials suitable for applications involving human contact.

## 2. Materials and Methods

CS with low-molecular-weight chitosan (50.000–190.000 Da based on the viscosity) with a degree of deacetylation of ≥75% and lot number BCCN2877, itaconic acid (IT) (lot number SDBB3434), and glacial acetic acid (lot number MKCX6123) were sourced from Sigma-Aldrich (St. Louis, MO, USA). Corn starch (S) (lot number S1810801) with a molecular weight of 692.65 g/mol was supplied by Everest Ltd. (Moscow, Russia). Carbon black was obtained from Balausa Firm LLP (Kyzylorda, Kazakhstan). Its specifications included a carbon content of 39.5%, particle size of ≤10 microns for 90% of particles, bulk density of 430 kg/m^3^, specific surface area of 79.9 m^2^/g, dibutyl phthalate absorption of 90 cm^3^/100 g, and moisture content of 0.5%. X-ray spectral analysis of the carbon black sample confirmed its elemental composition as follows: Na_2_O: 0.40%; MgO: 0.34%; Al_2_O_3_: 7.74%; SiO_2_: 40.36%; P_2_O_5_: 0.60%; K_2_O: 1.66%; CaO: 1.30%; TiO_2_: 0.37%; MnO: <0.10%; V_2_O_5_: 0.44%; BaO: 0.48%; SO_3_: 2.33%; Fe_2_O_3_: 2.45%; and p.p.p: 41.53%.

### 2.1. Synthesis of Chitosan-g-Itaconic Acid (CS-g-IT)

The graft copolymerization process was carried out in a 250 mL three-neck flask fitted with a thermometer, mechanical stirrer, and reflux condenser. Initially, chitosan was dissolved in 1% (*v*/*v*) acetic acid with continuous stirring at 85 °C. Once fully solubilized, the temperature was carefully maintained throughout the process. A freshly prepared potassium persulfate solution (0.1 g in 10 mL distilled water) was then introduced, followed by the gradual addition of itaconic acid (IT) in a dropwise manner. The polymerization was carried out for 2 h, followed by an additional 20 min of stirring at ambient temperature. The resulting copolymer was precipitated by adding 250 mL of isopropanol and then filtered and washed thoroughly to remove any unreacted low-molecular-weight chitosan [24,25]. To remove the homopolymer, the precipitate was refluxed in 20 mL of N, N-dimethylformamide (DMF) for 24 h. The final grafted product was dried under a vacuum at 60 °C. The specific ratios of chitosan to itaconic acid in the synthesized P(CS-g-IT) are listed in Table 1.

### 2.2. Synthesis of the Blend Copolymer P[(CS-g-IT)-b-S]

The blending process was carried out in a three-neck flask equipped with a mechanical stirrer and maintained under a nitrogen atmosphere. The grafted copolymer P(CS-g-IT) was initially dissolved in a 1% (*v*/*v*) acetic acid solution with constant stirring at 60 °C until complete dissolution was achieved. In a separate step, starch (S) was dissolved in 50 mL of distilled water and then gradually introduced into the flask containing the graft copolymer solution. The mixture was then heated to 95 °C and stirred at 600 rpm for 4 h, ensuring complete homogenization of the starch with the grafted copolymer solution. The blend copolymer’s mix ratios of the grafted copolymer P(CS-g-IT) and starch (S) are detailed in Table 1.

### 2.3. Film Formation

The grafted copolymers P(CS-g-IT) and blended copolymer P[(CS-g-IT)-b-S] were combined with 5% glycerol and heated at 50 °C for 30 min until homogeneous mixtures were achieved, which were designated as G and B, respectively. Additionally, variants of the blended copolymer P[(CS-g-IT)-b-S] were prepared by mixing 5% glycerol with varying proportions of carbon black (1%, 3%, and 7%), and these were labeled as CB, as shown in Table 1. The GG and CB films were prepared using the casting solution technique in thoroughly cleaned glass dishes. Aqueous dispersions of GG or CB were poured onto smooth glass plates and left to dry under ambient conditions (25 °C, 60% relative humidity) for seven days. After the initial drying, the films were placed in a ventilated oven at 60 °C for 12 h. To confirm complete moisture removal, film weights were recorded repeatedly—no fewer than three times—until a stable mass was observed [26,27]. After drying, the films were kept in a desiccator at room temperature until further testing and analysis. Throughout the evaluation of the effects of IT, starch, and carbon black on film properties, the chitosan concentration was held constant.

### 2.4. Measurements

#### 2.4.1. Fourier-Transform Infrared (FTIR) Spectroscopy

FTIR was carried out using a Bruker Tensor 37 instrument, scanning within the spectral range of 4000 to 400 cm^−1^.

#### 2.4.2. Viscosity Analysis

The viscosity (η) of the resulting dispersions was measured at 25 °C using a Brookfield viscometer (Model LVTDV-II) operating at a shear rate of 100 s^−1^. Viscosity values were determined by averaging the results from three separate measurements.

#### 2.4.3. Water Contact Angle Measurements

Water contact angle measurements were carried out with a CAHN DCA-322 instrument under standardized conditions (25 °C, droplet velocity of 100 µm/s). Water droplets were applied to the film surface using a microsyringe and their profiles were analyzed from the monitor to determine the angle. To ensure precision, measurements were taken from three distinct areas on each film sample dimensions 100 × 100 × 50 mm.

#### 2.4.4. Scanning Electron Microscopy Analysis (SEM)

The microstructure of hydrophilic polymers was examined through SEM using a Carl Zeiss SMT apparatus (Oberkochen, Germany). SEM imaging was conducted at an acceleration voltage of 15 kV. Samples with side lengths ranging from 5 to 10 mm were sectioned and mounted on aluminum stubs using double-sided carbon conductive tabs, then sputter-coated with a 1.3 nm thick layer of platinum prior to SEM analysis.

#### 2.4.5. Thermogravimetric Analysis (TGA)

TGA was carried out using a METTLER TOLEDO TGA/SDTA851e apparatus with a temperature accuracy of ±0.5 °C and a weight accuracy of ±0.01 mg under a nitrogen atmosphere at a purge rate of 50 mL/min. Approximately 8 mg of sample with a thickness of 1.0 mm was used for each experiment. The tests were conducted at a heating rate of 10 °C per minute over a temperature range of 20–600 °C. TGA data were used to determine the polymers’ thermostability and degradation temperature.

#### 2.4.6. The Tensile Strength and Elongation at Break

The tensile strength and elongation at the break of the films were evaluated by ASTM D 882-91 [28], employing an MTS 10/M tensile testing apparatus with a crosshead speed of 50 mm/min. Tensile tests were conducted on films and sheet materials less than 1.0 mm thick using a 1 kN load cell. The results were averaged from at least three independent replicates. Additionally, the thermostability and degradation temperature of the polymers were confirmed through TGA analysis. The tensile and elongation measurements were obtained as the average of three independent replicates.

### 2.5. Biodegradable Tests

To study the biodegradation of the films, three samples per film were buried in regular soil inside a plastic container, following the procedures reported by the authors [29,30]. Two soil types were utilized: one collected from the courtyard of Al-Faraby Kazakh National University in Almaty, Kazakhstan, and dry soil. The films were buried for 80 days in both moist and dry soil environments and their biodegradability was evaluated. The solubility of the films was analyzed by measuring the amount of dry matter dissolved after immersion in water at varying temperatures (30, 35, 40, and 45 °C). These values were subsequently compared to the original dry weight of the films prior to immersion. The degree of solubility was calculated using the following formula:

% Solubility = [(m − m_0_)/m] ×100

where m and m_0_ represent the weights of the films before and after immersion, respectively.

## 3. Results and Discussion

### 3.1. FTIR Spectra

**Figure 1:**
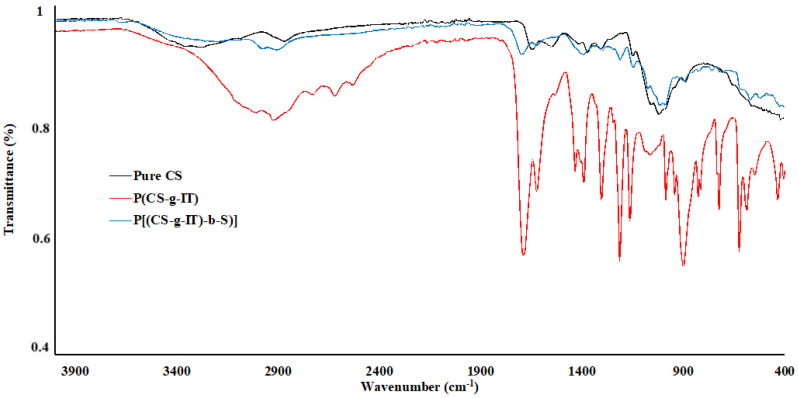
IR spectra of pure CS, P(CS-g-IT), and P[(CS-g-IT)-b-S)].

**Scheme 1:**
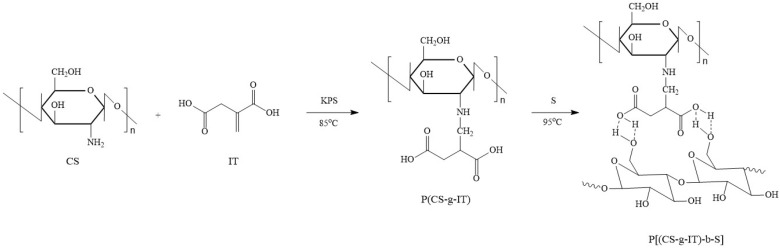
A schematic reaction of P(CS-g-IT) and P[(CS-g-IT)-b-S].

The FTIR spectrum of chitosan, illustrated in Figure 1, showed distinct bands indicative of its molecular structure. The broad band spanning 3205–3421 cm^−1^ highlighted N-H and O-H stretching, accompanied by intramolecular hydrogen bonding. The absorption bands at 2907 and 2862 cm^−1^ corresponded to symmetric and asymmetric C-H stretching, respectively, similar to those observed in characteristic polysaccharides [31,32,33]. Residual N-acetyl groups were evidenced by bands at 1640 cm^−1^ (C=O stretching of amide I) and 1373 cm^−1^ (C-N stretching of amide III). Notably, the expected band at 1550 cm^−1^ for N-H bending of amide II was absent, possibly masked by overlapping signals, while the 1548 cm^−1^ band reflected the N-H bending of the primary amine [34]. Additionally, CH_2_ bending and CH_3_ symmetrical deformations manifested as bands at 1416 and 1370 cm^−1^. The asymmetric stretching of the C-O-C bridge was identified at 1148 cm^−1^, and bands at 1020 and 1016 cm^−1^ denoted C-O stretching. These findings align well with previously documented chitosan spectra [26,27]. In the P(CS-g-IT) spectrum (Figure 1), new peaks appeared at 930–900 cm^−1^ and 1398 cm^−1^, characteristic of O-H bending (carboxylic group, IT). Peaks at 3250 cm^−1^ and 1691 cm^−1^ were attributed to the stretching vibrations of O-H and C=O, respectively, which were absent in pure chitosan, indicating the grafting of IT onto CS. Additionally, peaks observed at 2617 cm^−1^ and 2524 cm^−1^ corresponded to the C-H stretching band. The presence of NH in P(CS-g-IT) further confirmed the grafting polymerization by a peak at 1642 cm^−1^. The P[(CS-g-IT)-b-S] spectrum (Figure 1) revealed a decrease in the transmittance of hydroxy, carbonyl, and other groups, which was attributed to the blending of P(CS-g-IT) with S. The FTIR spectra of pure chitosan, P[(CS-g-IT)-b-S], and starch exhibited similarities due to their cyclic structures and the abundance of –OH bonds characteristic of saccharides, water, carboxylic acids, and antioxidants common to both chitosan and starch, as in the studies reported by Refs. [35,36]. The obtained biodegradable polymers are shown in Scheme 1. Due to hydrogen bonding, a very broad absorption band appeared in the 2500–3300 cm^−1^ region, overlapping with the C–H stretching vibrations of carbocyclic groups. Moreover, the O–H stretching band appeared broader and more intense, with a shift toward lower wavenumbers, generally in the range of 3200–3350 cm^−1^.

### 3.2. Viscosity

**Figure 2:**
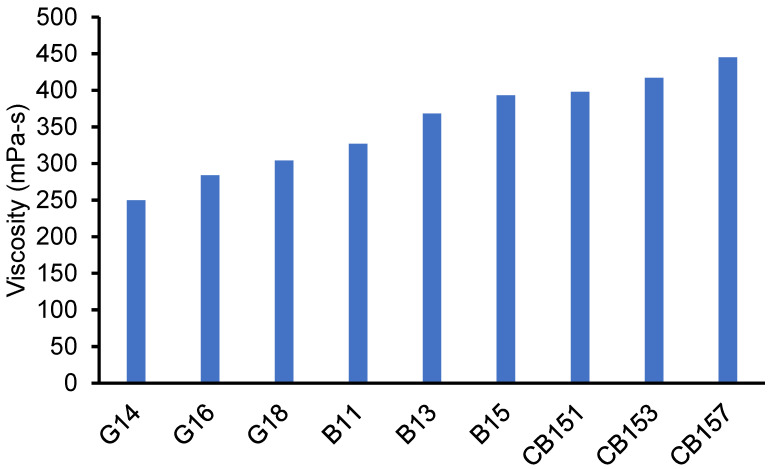
The viscosity values of biodegradable polymers are represented as follows: G for P(CS-g-IT), B for P[G18-b-S], and CB for B15 mixed with carbon black (C).

Figure 2 illustrates the effect of varying IT content on the viscosity of the grafted copolymer P(CS-g-IT). Viscosity increased as the IT content was raised from 4% to 8%, with the 4% IT content showing a viscosity of 250 mPa-s and the 8% IT content achieving a viscosity of 304 mPa-s. This increase in viscosity could be attributed to the formation of hydrogen bonds between the carboxylic groups of IT and the functional groups of CS [37]. The blending of starch with G18 led to an increase in viscosity by 7.5%, 21.1%, and 29.2% for starch contents of 1%, 3%, and 5% in P[(CS-g-IT)-b-S], respectively, as shown in Figure 2. This rise in viscosity could be attributed to the crosslinking interactions between starch and the carboxylic groups of IT. These findings aligned with expectations and are consistent with previously reported results by other authors [38], where the formation of hydrogen bonds and crosslinking between starch and acids resulted in increased polymer viscosity. The viscosity of the blended copolymer B15 increased when mixed with varying carbon black contents (1%, 3%, and 7%). As the carbon black content rose from 1% to 7%, the viscosity of the blended copolymer increased from 398 to 445 mPa-s, as shown in Figure 2. Similar findings were reported by Irmukhametova et al. [27] during their study on the characterization of biodegradable polymers based on polyvinyl alcohol, starch, and chitosan in the presence of carbon black.

### 3.3. Mechanical Properties

**Figure 3:**
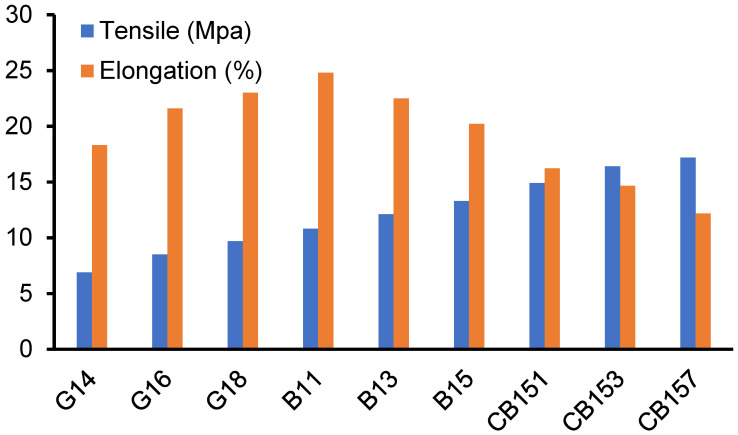
The tensile strength and elongation at the break of the biodegradable films are represented as follows: G for P(CS-g-IT), B for P[(G18)-b-S], and CB for B15 mixed with carbon black (C).

The impact of IT, starch, and carbon black contents on the tensile strength and elongation at break of the biodegradable films is shown in Figure 3. The findings demonstrate that incorporating IT into CS improved both tensile strength and elongation at break. Specifically, tensile strength rose by 23.1% as the IT content increased from 4% (G14) to 6% (G16) and by 40.5% with a further increase from 6% (G16) to 8% (G18). Similarly, elongation at break improved by 18.03% and 25.7% for IT contents increasing from 4% (G14) to 6% (G16) and then to 8% (G18), respectively. These improvements could be attributed to hydrogen bonding between IT and CS, along with crosslinking, which strengthened the films’ mechanical properties [38,39]. Blending starch with the grafted copolymer P[(G18)-b-S) (B) further enhanced the tensile strength: by 11.8% for 1% starch (B11), 24.7% for 3% starch (B13), and 37.11% for 5% starch (B15) compared to the grafted copolymer G18 (Figure 3). However, the elongation at the break of the films showed a decrease of 2.2% and 12.2% for 3% starch (B13) and 5% starch (B15), respectively, while increasing by 7.7% for 1% starch (B11) relative to G18. This additional improvement in tensile strength was due to stronger hydrogen bonding among IT, CS, and starch [26,39,40]. The incorporation of various carbon black contents into the blended copolymer P[B15-b-S] (CB) showed a 12.03% increase in tensile strength for 1% carbon black (CB151), but this value decreased to 11.2% for 3% carbon black (CB153) and 6.01% for 7% carbon black (CB157) in comparison with B15. The effect on elongation at break revealed reductions of 19.6%, 7.7%, and 12.3% for carbon black contents of 1% (CB151), 3% (CB153), and 7% (CB157), respectively.

**Figure 4:**
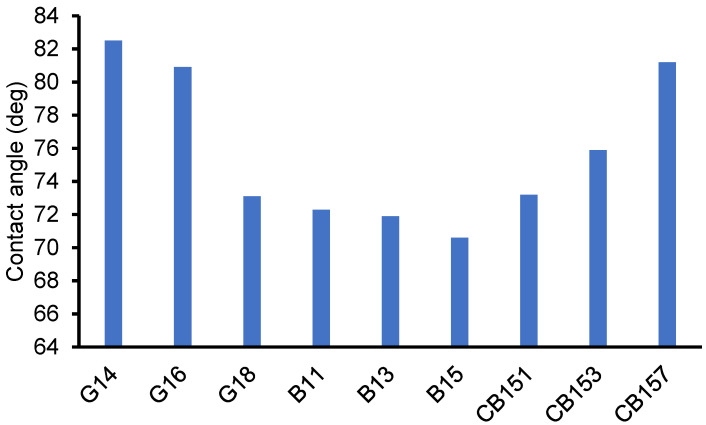
The contact angles of biodegradable films are represented as follows: G for P(CS-g-IT), B for P[(G18)-b-S], and CB for B15 mixed with carbon black (C).

Several factors can affect the hydrophilicity of polymeric materials, such as the chemical composition of polymers, molecular structure, additives, ingredients, and fillers used [41,42]. Figure 4 illustrates that IT enhanced the hydrophilicity of films due to the contribution of carboxy and hydroxyl groups in CS. This was evident from a decreasing contact angle as IT content increased from 82.5° for G14 (4% IT) to 73.1° for G18 (8% IT). While G18 blended with various contents of S, the contact angle decreased to 72.3° for B11 (1% S), 71.9° for B13 (3% S), and 70.6° for B15 (5% S). The decrease in contact angle was attributed to the effect of functional groups of S, including ether and hydroxy groups, on the film’s hydrophilicity. However, mixing carbon black in different ratios with blended copolymer B15 increased the contact angle from 70.6° for B15 (5% S) to 73.2° for CB151 (5% S + 1% carbon black), 75.9% for CB153 (5% S + 3% carbon black), and 81.2° for CB157 (5% S + 7% carbon black), as shown in Figure 4. The incorporation of carbon black reduced the water contact angle, indicating increased surface hydrophilicity. Samples containing carbon black showed lower contact angles than those without, suggesting improved hydrophilicity and potentially enhanced biodegradability. Additionally, the stress–strain curves, reported by Wei et al., 2018 [43], indicated the yield strength, ultimate tensile strength, and elongation at break, which were used to extract the corresponding mechanical property data.

### 3.4. Biodegradability Properties of P(CS-g-IT) and P[(CS-g-IT)-b-S)] Films

The biodegradation of the grafted copolymer (G): P(CS-g-IT), the blended polymers (B): P[(G18)-b-S)], and the blended polymers combined with varying amounts of carbon black (CB): B15 + carbon black was analyzed by immersing the films in soil for 80 days, and the findings are summarized in Table 2. The biodegradability of grafted copolymers with varying itaconic acid (IT) content was investigated. As shown in Table 2, the degradation times for grafted copolymers with 4% (G14), 6% (G16), and 8% (G18) IT content were 39, 34, and 32 days, respectively. This behavior was attributed to the presence of COOH, OH, and NH groups, which enhanced the hydrophilicity of the films by forming hydrogen bonds with water. Blending G18 with different amounts of S reduced the degradation time by 6.25% and 15% for blends containing 3% S (B13) and 5% S (B15), respectively, while 1% S had no significant effect. However, incorporating carbon black into the blended copolymer (B15) increased the degradation time. It rose by 22.22% for 1% carbon black (CB151), 40.7% for 3% carbon black (CB153), and 58% for 7% carbon black (CB157). The increased degradation time was due to carbon black reducing the film’s hydrophilicity in water.

**Figure 5:**
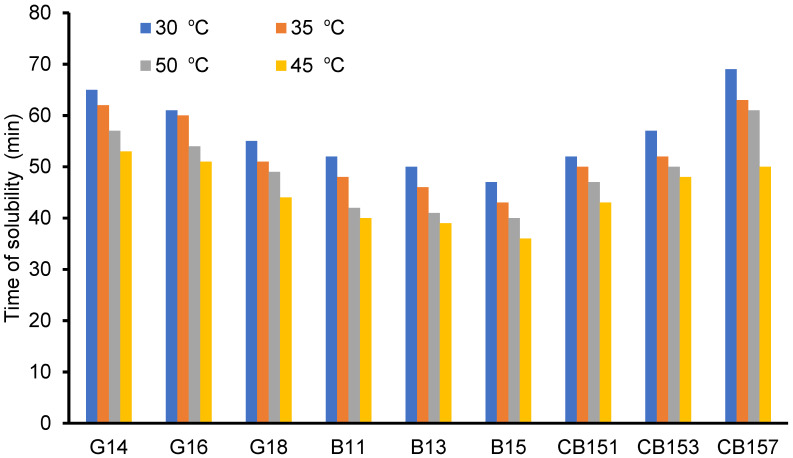
The effect of temperature on the biodegradability of films in the presence of IT, S, and carbon black. G for P(CS-g-IT)*,* B for P[(G18)-b-S]*,* and CB for B15 mixed with carbon black (C).

The solubility behavior of the grafted and blended biodegradable polymer films—including those with added carbon black—was evaluated under various temperatures and exposure times, as presented in Figure 5, to examine the effects of itaconic acid (IT), starch (S), and carbon black. The findings revealed that as the temperature rose from 30 °C to 50 °C, the solubility of the biodegradable films correspondingly increased. Specifically, the grafted polymer containing 8% IT (G18) reduced solubility time by 15.3% and 16.9% compared to the grafted copolymer with 4% IT (G14) at temperatures of 30 °C and 50 °C, respectively. In contrast, the blended biodegradable polymer with 5% S (B15) achieved an 8% and 18.1% reduction in solubility time compared to G18, which contained no S, at temperatures of 30 °C and 50 °C, respectively. Additionally, the incorporation of 7% carbon black into the blended copolymer (B15), resulting in formulation CB157, increased solubility time by 22% and 14% relative to B15 without carbon black at temperatures of 30 °C and 50 °C. The observed reduction in solubility time was primarily attributed to the roles of IT and S in enhancing water interaction with the biodegradable films, which exceeded the impact of carbon black.

### 3.5. Thermogravimetric Analysis (TGA)

**Figure 6:**
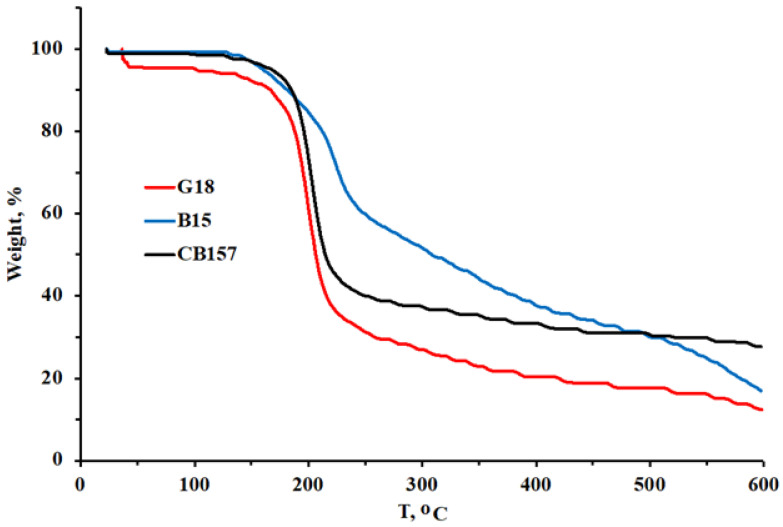
TGA thermogram of the biodegradable polymers G18, B15, and CB157.

Thermogravimetric analysis (TGA) is employed to evaluate a material’s thermal stability by monitoring weight changes relative to temperature and time. These weight variations result from structural and compositional changes in polymers due to processes such as decomposition and oxidation. Figure 6 presents the TGA curves for the grafted copolymer (G18), blended copolymer (B15), and blended copolymer with added carbon black (CB157), with the corresponding data summarized in Table 3. Thermal measurements were conducted across a temperature range of 20 °C to 600 °C, revealing that the decomposition of G18, B15, and CB157 occurred in three distinct stages, as outlined in Table 3. In the first stage, G18 showed a weight loss of 7.7% between 36.7 °C and 161.6 °C. For B15, the weight loss was 3.4%, occurring within the range of 23.3 °C to 153.1 °C. Similarly, CB157 experienced a weight loss of 5.6% between 23.8 °C and 174.6 °C. These initial weight losses were attributed to the evaporation of water and moisture [26,27,39]. The second stage of thermal decomposition for G18, B15, and CB151 occurred within the temperature ranges of 161.6–323.1 °C, 153.1–327.4 °C, and 174.6–325.3 °C, respectively. During this stage, weight losses of 65.9%, 48.9%, and 57.4% were observed, attributed to the decomposition of CS, IT, S, and carbon black [40,41]. The third stage of decomposition for G18, B15, and CB157 occurred within the temperature range of 320–600 °C, resulting in weight losses of 23.9%, 19.4%, and 8.6%, respectively. These losses were attributed to the breakdown of CS, carbon black, and starch [44,45].

### 3.6. Scanning Electron Microscopy (SEM)

**Figure 7:**
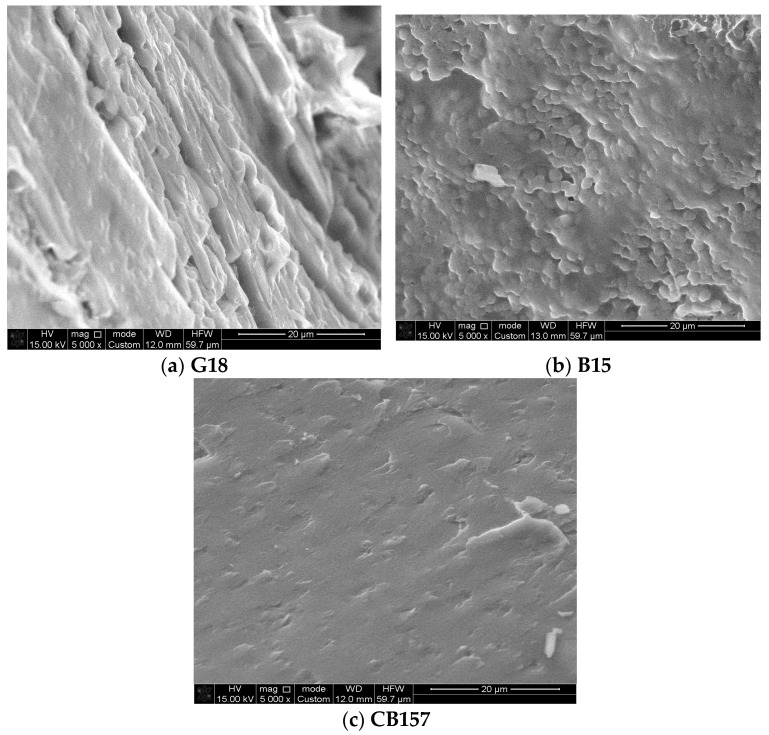
SEM images of the prepared films of (**a**) P(CS-g-IT) (G18), (**b**) P[(G18)-b-S] (B15), and (**c**) P[(G18)-b-S] + carbon black (CB157).

Scanning electron microscopy (SEM) was employed to examine the surface morphology of the synthesized P[chitosan–g–itaconic acid] composite films. Figure 7 displays the SEM micrographs of the films: P(CS-g-IT), P[(CS-g-IT)-b-S], and P[(CS-g-IT)-b-S] with carbon black. As seen in Figure 7a, the P(CS-g-IT) film exhibited a uniform and pore-free surface, likely due to the absence of a cross-linking agent. As shown in Figure 7b, the P[(CS-g-IT)-b-S] film revealed a smooth, flake-like, layered structure with minimal voids and maintained a uniform texture, confirming successful synthesis in the absence of a cross-linker. Figure 7c illustrates the morphology of the film containing carbon black, where the fractured surface appeared more consolidated, attributed to the carbon black content. Minor surface cavities observed in all samples (a–c) were likely due to the aggregation of CS, itaconic acid, starch, and carbon black. Notably, the composite containing carbon black displayed a finer and more uniform surface compared to its counterpart without carbon black. This effect may have been due to the presence of carbon black, which enhanced the film’s biodegradability by introducing small holes. It also enhanced the dispersion of itaconic acid within the chitosan matrix and thus improved the interfacial interactions between chitosan and itaconic acid particles.

## 4. Conclusions

Biodegradable polymers were synthesized using grafting polymerization of chitosan (CS) with various composition ratios of itaconic acid (IT) and the blending polymerization of P(CS-g-IT) with different proportions of starch (S). Additionally, the blended copolymer, P[(CS-g-IT)-b-S], was mixed with varying amounts of carbon black (C). The incorporation of starch into the grafted copolymer enhanced the biodegradability rate of the material compared to IT and carbon black. The results demonstrated that the addition of carbon black to the blended polymer mixture improved thermal stability and biodegradability but reduced the mechanical properties of the polymer more significantly than IT. Among the tested formulations, the blended copolymer containing 1% grafted copolymer and 5% starch exhibited the best mechanical properties and shortest degradation time. This formulation (B15) is therefore considered the most stable and effective biodegradable polymer blend for potential applications. This is followed by the grafted copolymer with IT at a 5:1 ratio and the blended copolymer with 7% carbon black.

## Figures and Tables

**Table 1:** The blend copolymers based on grafted copolymers P(CS-g-IT) and S and carbon black with different ratios.

Samples/ Ingredients	P(CS-g-IT) Wt. %		Samples/ Ingredients	P[(G18)-b-S] Wt. %		Samples/ Ingredients	P[(B15)-b-S] + Carbon Black (C), Wt. %	
	CS	IT		G18	S		B15	C
G14	1	4	B11	1	1	CB151	1	1
G16	1	6	B13	1	3	CB153	1	3
G18	1	8	B15	1	5	CB157	1	7

**Table 2:** The effect of ground soil on the biodegradability of the films.

Time of Film Weight Until 90% (Days)					
Samples	P(CS-g-IT)	Samples	P[(G18)-b-S]	Samples	CB (B15 + C)
G14	39	B11	32	CB151	33
G16	34	B13	30	CB153	38
G18	32	B15	27	CB157	41

**Table 3:** Thermal properties of grafted copolymer (G18), blended copolymer (B15), and blended copolymer mixed carbon black (CB157).

Samples No.	Ingredients	Weight Loss (%)	Temperature, °C
G18	P(CS-g-IT) (1:8)	7.69	36.7–161.59
		65.88	161.59–323.1
		23.86	323.1–598.1
B15	P[(G18)-b-S] (1:5)	3.4	23.27–153.11
		48.89	153.11–327.41
		19.39	327.41–597.89
CB157	P[(B15) + C] (1:7)	5.56	23.75–174.63
		57.36	174.63–325.27
		8.58	325.27–597.57

## Data Availability

Raw data will be provided upon request.
